# Long Term Transcriptional Reactivation of Epigenetically Silenced Genes in Colorectal Cancer Cells Requires DNA Hypomethylation and Histone Acetylation

**DOI:** 10.1371/journal.pone.0023127

**Published:** 2011-08-04

**Authors:** David Mossman, Rodney J. Scott

**Affiliations:** 1 Discipline of Medical Genetics, School of Biomedical Sciences, Faculty of Health, University of Newcastle, Callaghan, New South Wales, Australia; 2 Hunter Medical Research Institute, New Lambton Heights, New South Wales, Australia; 3 Division of Genetics, Hunter Area Pathology Service, John Hunter Hospital, Newcastle, New South Wales, Australia; Oregon State University, United States of America

## Abstract

Epigenetic regulation of genes involves the coordination of DNA methylation and histone modifications to maintain transcriptional status. These two features are frequently disrupted in malignancy such that critical genes succumb to inactivation. 5-aza-2′-deoxycytidine (5-aza-dC) is an agent which inhibits DNA methyltransferase, and holds great potential as a treatment for cancer, yet the extent of its effectiveness varies greatly between tumour types. Previous evidence suggests expression status after 5-aza-dC exposure cannot be explained by the DNA methylation status alone.

**Aim:**

We sought to identify chromatin changes involved with short and long term gene reactivation following 5-aza-dC exposure. Two colorectal cancer cell lines, HCT116 and SW480, were treated with 5-aza-dC and then grown in drug-free media to allow DNA re-methylation. DNA methylation and chromatin modifications were assessed with bisulfite sequencing and Chromatin Immuno-Precipitation analysis.

**Results:**

Increased H3 acetylation, H3K4 tri-methylation and loss of H3K27 tri-methylation were associated with reactivation. Hypermethylated genes that did not show increased acetylation were transiently expressed with 5-aza-dC treatment before reverting to an inactive state. Three reactivated genes, CDO1, HSPC105 and MAGEA3, were still expressed 10 days post 5-aza-dC treatment and displayed localised hypomethylation at the transcriptional start site, and also an increased enrichment of histone H3 acetylation.

**Conclusions:**

These observations suggest that hypomethylation alone is insufficient to reactivate silenced genes and that increased Histone H3 acetylation in unison with localised hypomethylation allows long term reversion of these epigenetically silenced genes. This study suggests that combined DNA methyltransferase and histone deacetylase inhibitors may aid long term reactivation of silenced genes.

## Introduction

The human genome contains approximately 3 billion base pairs of DNA [1] that require strategic packaging into a compact, yet dynamic structure. Condensation is achieved with the supercoiling of ∼147 bp DNA around an octamer of histone proteins (two copies of each H2A, H2B, H3 and H4) to form a nucleosome [2] which impedes accidental gene expression and increases the dependence of transcriptional activators [3]. Transcriptional repression can be mediated by DNA methylation and is assisted by extensive modifications at highly conserved lysine residues on the tails of histone proteins. Lysine acetylation facilitates transcription by weakening the association of the histone and DNA [4] and allows transcription factor binding [5]. Lysine methylation is more complex and can be associated with both active and repressed regions of DNA, and may be present in mono-, bi-, and tri-methylated forms [6]. For instance, trimethylation of histone H3 lysine 4 (H3K4me3) is an active mark [7] whilst methylation of H3K9 and H3K27 appears at transcriptionally silent gene promoters [7], [8].

Aberrant epigenetic silencing of genes can initiate malignancy and frequently appears in addition to genetic alterations, contributing to disease progression in several forms of cancer [9], [10], [11]. In addition, aberrant hypomethylation of proto-oncogenes can lead to their activation [12], [13] Reduced expression of numerous genes due to epigenetic silencing correlates with poor prognosis in many forms of malignancy such as lung [14], melanoma [15], breast [16], gastric [17] and colon [18]. Rare instances of soma-wide mono-allelic methylation of MLH1 have been shown to arise via germline transmission [19]. In addition, heritable copy-number variations can result in transcriptional read through and in-*cis* methylation when adjacent to key genes [20]. These mechanisms offer an explanation of why some families are at a higher risk of disease development despite not carrying an underlying genetic mutation of crucial genes. Individuals within such families could benefit from early detection of aberrant epigenetic marks at genes which confer an elevated risk of a particular disease. With an increasing awareness of epigenetic abnormalities in disease, counteracting these changes with methyltransferase inhibitors such as 5-aza-2′-deoxycytidine (5-aza-dC) would appear to be a potentially effective treatment. In reality, this treatment is not effective in a specific group of tumour types [21], which may be due to reactivated genes reverting to a silenced state upon cessation of treatment.

We have previously identified the reactivation of numerous genes in colorectal cancer cell lines following treatment with the demethylating agent 5-aza-dC [22]. Upon removal of the drug and ten days of growth, some of these genes remained highly expressed, suggesting a reversal of the transcriptional status of these genes. Although reduced by 5-aza-dC, the changes in DNA methylation did not correlate with the levels of expression in the group of genes analysed, indicating other epigenetic modifications were controlling transcription. The genes selected for analysis were examined due to their involvement in a range of tumour types and possible use as biomarkers in these tumours [23], [24], [25], and/or due to their strong re-expression and pattern of gene expression following 5-aza-dC in colorectal cancer cells [22]. CDKN2A was chosen specifically as it is frequently repressed in colorectal cancer tumours [26]. These genes may represent important genes in the epigenetic development of a number of tumour types. In this study we have characterised the changes of DNA methylation and chromatin state which allow for either a long or short term reactivation of expression following 5-aza-dC exposure.

## Methods

### Cell Culture

Triplicate cultures of HCT116 and SW480 cells were grown in DMEM media supplemented with 10% foetal calf serum (Sigma-Aldrich, St Louis, MO, USA) at 37°C and 5% CO_2_. Cells were treated with 5-aza-2′-deoxycytidine (Sigma-Aldrich) as previously described [22]. DNA and RNA were extracted from untreated cells, 5-aza-dC treated cells (72 h of treatment), and at 4 and 10 days after cessation of treatment (Day 4 and 10 of re-methylation). The cells were originally obtained from the ATCC and were authenticated using the Identifiler DNA identification kit (Applied Biosystems, Foster City, CA, USA) according to manufacturer's instructions.

### Global methylation

Global methylation was assessed as described previously [22]. Briefly, 50 µg of DNA was enzymatically digested with Nuclease P1 (US Biological, Swampscott, MA, USA) followed by chromatographic separation on a Varian Star Chromatography workstation with a Supelcosil LC-18-DB column (Sigma-Aldrich). Absorbance was monitored at 278 nm and peak areas were quantified with Star Reviewer Software (Varian, Palo Alto, CA, USA). The 5-methylcytosine content was expressed as a percentage of the total cytosine pool after correction for extinction co-efficients.

### Bisulfite Sequencing

DNA was converted in duplicate using a Qiagen Epitect Bisulfite conversion kit (Qiagen, Valencia, CA, USA) using 2 µg of phenol-chloroform purified DNA. Samples were eluted in 30 µL of elution buffer and an aliquot was diluted 1∶3 prior to PCR and stored at 4°C, whilst the remaining fraction was stored at −20°C. CpG islands surrounding the transcription start site of genes were targeted in PCR analysis using the primers listed in Table S1. Sequencing reactions were performed in duplicate and were analysed on an ABI 3730 sequencer. Data analysis was carried out using Sequence Scanner software (Applied Biosystems). The percentage methylation at each CpG was determined by dividing the cytosine peak by the combined heights of the cytosine and thymine peaks as described previously [27].

### Real Time PCR Analysis of gene expression

RNA was converted to cDNA using Superscript II (Invitrogen) and random primers (Promega) according to manufacturer's instructions. Reactions were performed in triplicate using primers listed in Table S1, 2× SYBR Green (Applied Biosystems) in an ABI PRISM 7500 PCR machine (Applied Biosystems). C_T_ values were determined automatically by Sequence Detection Software version 1.4 and final calculations were expressed as fold differences compared to B-actin using the ΔΔC_T_ method. Genes with undetected expression were assigned a C_T_ value of 40. Error bars in expression figures represent the standard error.

### Chromatin Immunoprecipitation (ChIP) and Analysis

Briefly, the crosslinking of DNA with protein and cell lysis was performed using the EZ-Magna ChIP A Kit (Upstate/Millipore) according to the manufacturer's instructions. Sonication was performed using 8×30-second cycles at 60% duty cycle in an ice bath and samples were further cooled for 30 seconds in between sonication cycles. Chromatin Immunoprecipitation was performed as previously described [28] with slight modifications. Antibodies were obtained from Upstate (Catalog numbers; α-H3Ac 06-599, α-H3K4me3 07-473, α-H3K9me3 17-625, α-H3K27me3 17-622) with the exception of the Rabbit IgG non-specific antibody from Santa Cruz Biotechnology (Catalog number SC2027). Antibody quantities per reaction were determined in preliminary experiments and were 5 µL for α-acetyl H3, 5 µL for α-H3K4me3, 4 µL for α-H3K9me3, 4 µL for α-H3K27me3. 5 µL of Rabbit IgG was added to negative control samples. Cross links were reversed with the addition of 20 µL Proteinase K (Promega) and incubated at 62°C for 3 h with shaking. Recovered DNA was then purified using a PCR clean up kit (Qiagen, Valencia, CA, USA).

### Real time PCR analysis of immunoprecipitated chromatin

DNA was quantified using the DNA Quantitation System (Promega) according to the manufacturer's instructions, and measurements were taken using a TD 20/20 luminometer (Turner Designs, Sunnyvale, CA, USA). The real-time PCR reactions were performed using 200 ρg of DNA template with SYBR Green 2× mastermix (Applied Biosystems) and primers listed in Table S1. Reactions were performed in triplicate and carried out using an ABI PRISM 7500 PCR machine (Applied Biosystems). C_T_ values were determined automatically by Sequence Detection Software version 1.4 (Applied Biosystems) and the final values were expressed as a percentage of the input fraction. Error bars in chromatin change figures represent the standard error.

### Statistical Analysis

Standard deviations were calculated and a T-test was employed to compare expression levels and histone modification levels in drug treated cells against untreated cells. *P*-values less than 0.05 were considered to be statistically significant.

## Results

### Genomic DNA Methylation with 5-aza-dC treatment

**Figure 1:**
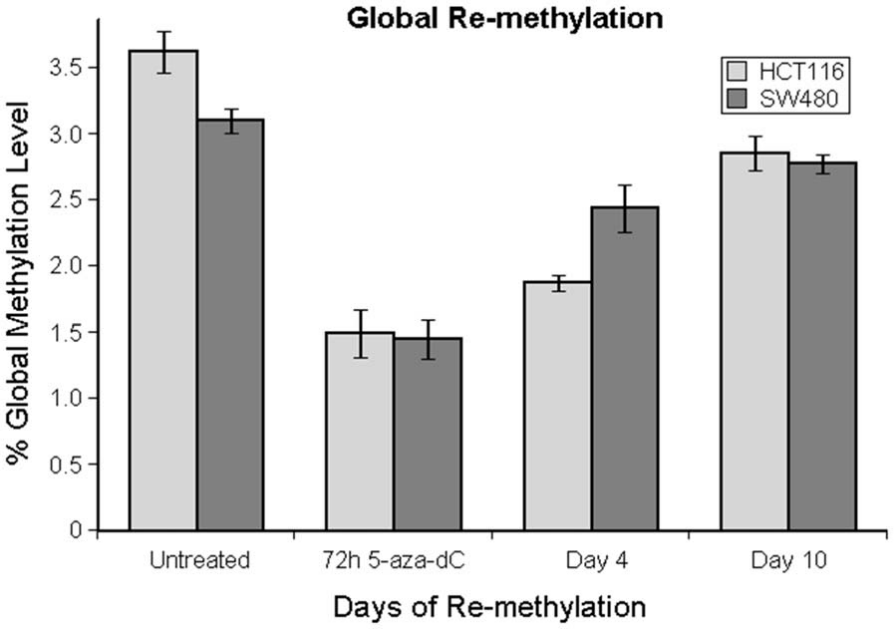
Genome wide methylation levels and 5-aza-dC treatment. Genomic methylation levels decreased significantly in both cell lines following 5-aza-dC exposure. Genomic methylation levels were gradually restored over the next ten days of drug-free growth where they were approaching pre-drug treatment levels.

Global methylation levels decreased following 5-aza-dC treatment by 53% and 59% in the HCT116 and SW480 cell lines respectively (figure 1). This represents a significant decrease compared with cells that were mock treated which did not undergo demethylation (HCT116 p-value = 0.003, SW480 p-value = 0.017). Continued incubation of cells for a further ten days after treatment in drug free media allowed DNA re-methylation and genomic levels increased, but did not return to the same level as observed prior to treatment in this period.

### Gene Specific methylation and re-expression with 5-aza-dC treatment

Using genome wide expression arrays we have previously identified patterns of gene expression following 5-aza-dC treatment [22]. The same genes were again examined in this study to allow characterisation of histone modifications. In this experiment, expression was determined with quantitative PCR and genes were then classified into five categories; ‘always-expressed’ (expression detected at all time points), ‘up-regulated’ (two-fold in expression after 5-aza-dC treatment), ‘long term reactivated’ (undetected in untreated cells, but expressed after treatment as well as four and ten days after drug exposure (based on a C_T_ value of 40 for undetectable transcripts)), ‘short term reactivated’ (same as ‘long term reactivated’ with the exception of day four and/or ten expression which was required to be <100-fold above the level of untreated cells), or ‘other’ (any other pattern of gene expression).

**Figure 2:**
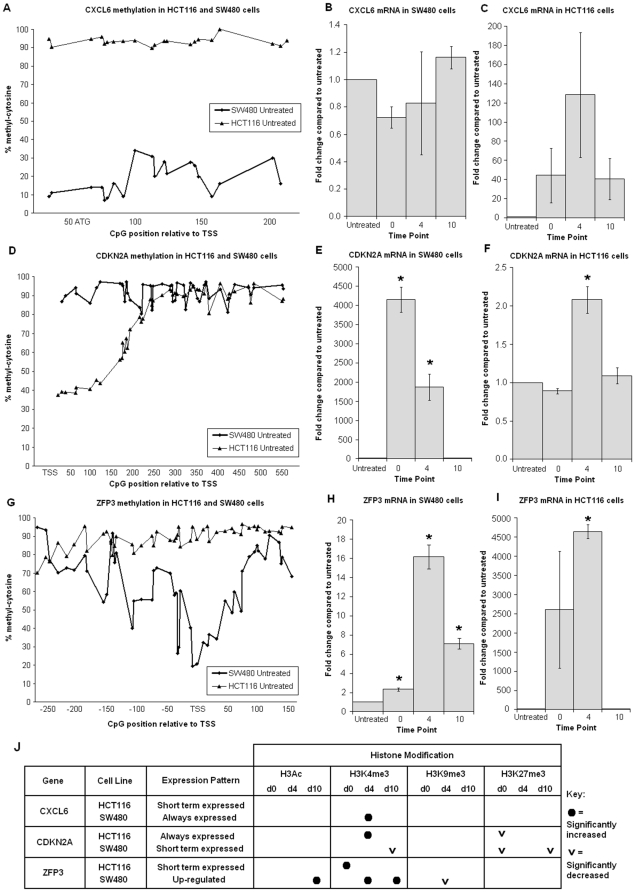
CpG island methylation of short term reactivated genes with respect to different expression types. **A** – CXCL6 CpG island methylation; short term v always-expressed. (**B**) - SW480 cells displayed hypomethylation and CXCL6 was expressed at all time points. (**C**) - Uniform hypermethylation of the HCT116 cell line was associated with a short term reactivation of expression. **D, E, F** – CDKN2A CpG Island methylation; short term v constant expression. SW480 cells display CDKN2A hypermethylation and were temporarily re-expressed, whilst in HCT116 cells CDKN2A is ∼50% methylated at CpG sites near the TSS, and was expressed at all time points. Asterisks denote significant change compared to untreated cells. **G, H, I** – ZFP3 CpG Island methylation; short term v up-regulated expression. SW480 cells showed hypomethylation at CpG sites near the TSS and expression was up-regulated after 5-aza-dC treatment. ZFP3 remains hypermethylated in HCT116 cells and was temporarily reactivated with 5-aza-dC treatment . **J** – Significant changes to histone modifications compared with untreated cells (p<0.05).

**Figure 3:**
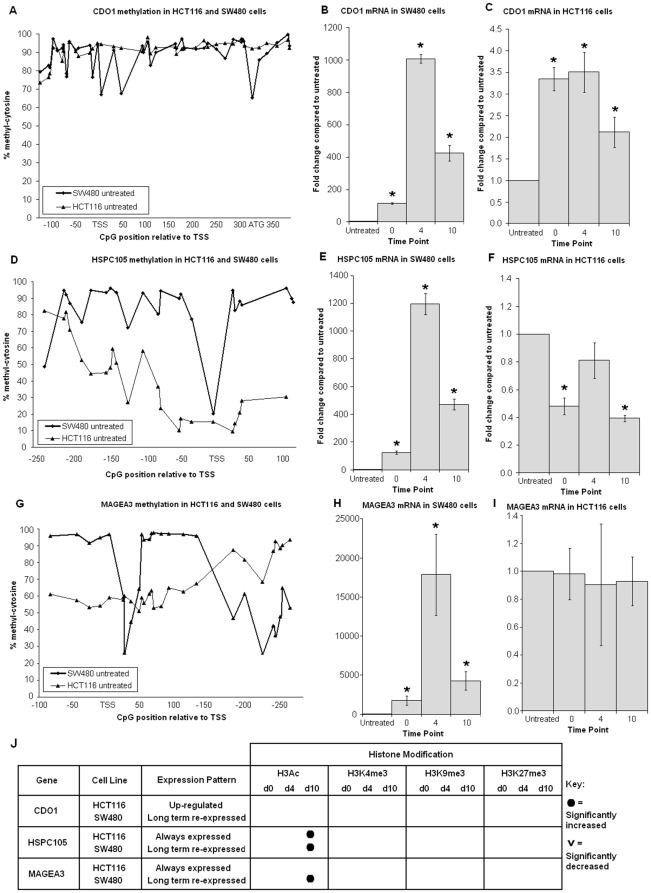
Promoter CpG Island methylation of long term reactivated genes. **A, B, C** – CDO1 CpG Island methylation; long term v short term expressed. Sequencing analysis revealed CpG sites near the TSS in SW480 cells have lower methylation and displayed long term expression compared with HCT116 cells which are uniformly hypermethylated at CDO1 and experienced an up-regulated pattern of expression. **D, E, F** - HSPC105 CpG Island methylation; always-expressed v long term expressed. The SW480 cell line shows localised hypomethylation at the TSS and can remain expressed ten days post treatment. The HCT116 cell line is hypomethylated at the HSPC105 promoter and is continually expressed. **G, H, I** – MAGEA3 CpG Island methylation; always-expressed v long term re-expressed. SW480 cells show localised hypomethylation at the TSS and are expressed ten days post treatment. HCT116 cells show ∼50% methylation at the TSS and MAGEA3 is expressed at all time points. **J** – Significant changes to histone modifications compared with untreated cells (p<0.05).

The three genes which were reactivated for a short period only (CXCL6 and ZFP3 in HCT116 cells and CDKN2A in SW480 cells) were all hypermethylated across the assayed region of their CpG islands. The expression pattern of these genes was markedly different in the other of the two cell lines; here, these genes showed very little or low methylation at the transcription start site (TSS) and were either expressed continually or became up-regulated (figure 2). The genes which remained highly expressed after reactivation (CDO1, HSPC105, MAGEA3) displayed unique methylation profiles and featured a hypomethylated CpG site adjacent to the transcription start site (TSS) as shown in figure 3. The CpG island specific demethylation after treatment was 10–15% at most,, therefore only untreated methylation patterns are shown in Figure 2 and 3. Data for the four time points is shown in Figure S1 for the MAGEA3 gene which displayed the largest gene associated decrease in DNA methylation. An example of the direct sequencing chromatograms of MAGEA3 are shown in Figure S2.

Genes determined to be ‘always-expressed’ displayed a maximum of 50% methylation at the TSS which suggests mono-allelic methylation, and up-regulated genes displayed varying patterns of methylation. The MLH1 gene was expressed in both cell lines and methylation was not detected in the TSS associated CpG island (data not shown). Conversely, a variant of DICER1 was not expressed in either cell line at any time point as determined by microarray analysis and the absence of its expression was confirmed by quantitative PCR. DICER1 is not associated with a CpG island, therefore bisulfite sequencing analysis was not performed. CDKN2A was expressed in HCT116 and showed partial methylation at the TSS. The corresponding region in SW480 cells was hypermethylated and expression was classified as short term reactivated after treatment.

Continued incubation of cells in drug free media allowed re-methylation of the DNA, which returned to original levels at promoter CpG islands. Gene expression was not necessarily affected by the return of promoter methylation however, and expression of the CDO1, HSPC105 and MAGEA3 genes in SW480 cells remained high ten days after 5-aza-dC treatment. These genes displayed unique CpG island methylation patterns that feature hypomethylated CpG sites adjacent to the TSS. As methylation levels at promoter CpG islands did not accurately reflect the expression of the genes studied, we sought to examine the patterns of histone modifications at the respective CpG islands that may account for high levels of expression.

### Chromatin modifications after 5-aza-dC exposure

Chromatin Immunoprecipitation and q-PCR revealed that Histone H3Ac and H3K4me3 were features associated with expressed genes such as GAPDH and MLH1 and repressed genes were associated with H3K9me3 and less frequently H3K27me3 which were absent from constitutively expressed genes.. ChIP results are summarised in Figure 2 and 3, with p-values listed in Table 1. Specific changes in chromatin modification are shown in Figures S3, S4, S5, S6.

**Table 1:** P-values for changes to histone modifications in comparison to untreated cells.

Gene	Cell line	H3Ac			H3K4me3			H3K9me3			H3K27me3		
		d0	d4	d10	d0	d4	d10	d0	d4	d10	d0	d4	d10
**CDKN2A**	**HCT116**	0.169	0.518	0.627	0.240	0.034*	0.076	0.724	0.209	0.339	0.019*	0.803	0.741
	**SW480**	0.519	0.338	0.515	0.801	0.846	0.047*	0.946	0.756	0.582	0.010*	0.245	0.034*
**CXCL6**	**HCT116**	0.372	0.271	0.156	0.083	0.052	0.052	0.154	0.784	0.355	0.068	0.620	0.592
	**SW480**	0.359	0.185	0.274	0.167	0.025*	0.063	0.223	0.076	0.113	0.238	0.086	0.184
**ZFP3**	**HCT116**	0.312	0.182	0.416	0.003*	0.269	0.219	0.588	0.049*	0.954	0.767	0.845	0.664
	**SW480**	0.464	0.271	0.035*	0.472	0.007*	0.031*	0.202	0.064	0.088	0.310	0.684	0.827
**CDO1**	**HCT116**	0.683	0.596	0.821	0.488	0.488	0.396	0.479	0.866	0.147	0.759	0.431	0.478
	**SW480**	0.280	0.162	0.080	0.129	0.403	0.823	0.879	0.509	0.826	0.354	0.141	0.199
**HSPC105**	**HCT116**	0.943	0.123	0.020*	0.365	0.499	0.691	0.343	0.682	0.059	0.807	0.310	0.725
	**SW480**	0.107	0.144	0.008*	0.271	0.115	0.522	0.328	0.421	0.274	0.359	0.288	0.988
**MAGEA3**	**HCT116**	0.314	0.254	0.267	0.934	0.382	0.248	0.992	0.600	0.670	0.969	0.686	0.261
	**SW480**	0.317	0.074	0.022*	0.073	0.091	0.165	0.304	0.826	0.427	0.897	0.334	0.285

Significant changes compared to untreated cells are marked with an asterisk (p = 0.05). Results are based on triplicate cell cultures and triplicate measurements for each time point.

Changes to the chromatin proteins following 72 h of exposure were dependant on the gene expression status. Genes with greater expression after treatment increased in H3K4me3, while H3Ac was only associated with genes reactivated for longer periods. Generally the repressive H3K27me3 marks were reduced after treatment, with the exception of CXCL6 gene. A comparison of histone modifications in short term reactivated genes revealed transient increase of histone H3K4me3 and decreased or stable level of trimethyl-histone H3 lysine 9 and 27. Despite this, transcription of these genes ten days post treatment was at a similar to untreated cells. The single most apparent difference between short and long term reactivated genes was the H3Ac modification. Genes deemed to be ‘long term reactivated’ revealed an increase of H3Ac, although not always reaching statistical significance, after treatment which persisted until ten days after drug treatment. There was also a trend in long term re-expressed genes where a reduction in H3K27me3 was observed The up-regulated genes (CDO1 in HCT116 cells and ZFP3 in SW480) and genes which were always expressed showed a gain of active chromatin marks and a loss of repressive histone modifications after 5-aza-dC exposure.

## Discussion

The epigenetic control of gene expression is mediated by DNA methylation and histone modifications. By altering the DNA methylation and up-regulating gene expression we can identify patterns of change in histone protein modifications that accompany the long and short term reactivation of epigenetically silenced genes. Of particular relevance to this study was the apparent transcriptional up-regulation of silenced genes that displayed less than 15% DNA demethylation, where histone modifications are likely to be involved in the regulation of gene expression in these instances.

### The effect of DNA methylation on gene expression

Regardless of the expression pattern during drug treatment, the extent of methylation of particular CpG islands remained relatively unchanged compared to genomic levels after 5-aza-dC exposure. This observation has been made previously, with methylation of repeat sequences likely to contribute to this discrepancy [22], [29]. Another study has shown that DNA methylation increases at promoters of non-expressed genes when inhibited by doxycycline in a tet-responsive promoter system [30]. Constitutively expressed genes were hypomethylated on both alleles, or exhibited CpG island methylation of 50% indicating mono-allelic methylation, such as the CDKN2A gene in HCT116 cells as previously shown [31]. Slight demethylation of promoter CpG islands was induced with 5-aza-dC, yet expression was not necessarily restricted in some genes when the methylation returned to original levels. High expression of long term reactivated genes appeared to be dependent upon pre-existing hypomethylation at the TSS regardless of whether the adjacent CpG sites were hypermethylated. This result indicates the pattern of methylation rather than the general level of methylation across the CpG island is crucial to re-activating silenced genes via interactions with other epigenetic factors. Hypomethylated CpG sites within hypermethylated promoters have been identified previously in the Oncostatin M receptor gene [27], but the effect of this hypomethylation on transcription was not examined. Why expression of the long term reactivated genes did not occur in the untreated cells which displayed a near identical methylation pattern may be explained by changes in the modifications on the histone proteins.

### The effect of histone modifications on gene expression

A snapshot of factors governing gene expression was achieved with the compilation of CpG island sequencing and chromatin immuno-precipitation results. Upon the reactivation of numerous genes and classification of expression, we could distinguish between the genes based on the methylation and chromatin changes present. Prior to treatment, transcriptionally inactive genes were characterised by higher levels of repressive modifications and lower levels of activating marks. Upon treatment with 5-aza-dC there was generally an increase to the levels of H3K4me3 and H3K9me3, whilst H3Ac increased only in some of the genes. Reductions to H3K27me3 occurred in genes that initially displayed this trait. With regards to chromatin modifications, it was during the drug free growth period that histone acetylation became the most distinguishable feature between short and long term reactivated genes. Long term reactivated gene promoters became increasingly associated with acetylation of histone H3 assisting with gene activation however only reached significant levels after ten days of drug free growth. Temporarily reactivated genes did not attract this modification despite a brief period of expression. It would therefore appear that the introduction of H3 acetylation is a crucial factor in reversing transcriptional status of an epigenetically silenced gene which is assisted by localised DNA hypomethylation. With the exception of H3K27me3 at CDKN2A in SW480 cells, long lasting changes to epigenetic modifications were not observed in temporarily reactivated genes.

The up-regulation of lowly expressed genes was associated with increased H3 acetylation and H3K4me3, such as the ZFP3 gene in SW480 cells. Methylation profiles such as this may indicate an intermediate between always-expressed genes and the long term reactivated genes. Co-existing active and repressive marks may allow a restricted level of transcription, suggesting the control of expression of these genes is dependent on equilibrium of both types of modifications.

In the genes examined in this study, the roles of histone H3 acetylation and H3K27me3 were apparent as activating and repressing marks respectively, however the effect of H3K4me3 and H3K9me3 did not appear sufficient to alter gene expression on a long term basis. Following 5-aza-dC exposure, H3K9me3 was frequently increased at expressed genes which concurs with recent findings that it can also be coupled with gene activation [29], [32], [33]. Similarly, H3K4me3 which associates with active regions of the genome was found at inactive genes, albeit at a reduced level. Observations of this nature highlight the dynamic nature of chromatin and possibly suggest an intermediate form of repression similar to bivalent chromatin surrounding developmental genes [34] or the effect of neighbouring chromatin that has been detected because of variations in shearing efficiency during sonication.

### Linking DNA methylation, chromatin modifications and gene expression

The patterns of expression of re-activated and up-regulated genes can be largely explained with the combination of promoter methylation analysis and chromatin immuno-precipitation assays. By observation and comparison of long and short term reactivated genes, our results show that genes with a localised hypomethylation at the TSS are more likely to experience an increase of histone H3 acetylation and remain expressed after 5-aza-dC exposure. Additionally we observed that specific gene expression was reactivated without major change in the associated CpG island methylation and this was independent of nearby hypermethylation within the same CpG island.

A sequence of events involved with epigenetic reactivation has been proposed by Litt *et al.* [35]. The authors state that reactivation of the HPRT gene required hemi-demethylation of the promoter, ‘opening’ of chromatin structure, transcription factor binding and assembly of the transcription complex prior to synthesis of the HPRT RNA. Based on our experiments, we can extend on this knowledge by suggesting that minor demethylation induced by 5-aza-dC and increased H3K4me3 allows initiation of transcription. The role of H3K9 trimethylation is not clear but may be associated with reactivation in some instances. A loss of repressive marks such as H3K27me3 can also further increase transcription. Although not examined here, it is possible MBD2 binding could be lost at this point which would no longer deter histone acetyltransferase from the region [36]. Transcription is prolonged if increased acetylation of histone H3 occurs, otherwise expression is transient and gene expression is likely to revert to an inactive state.

### Methyltransferase Inhibitors in treatment of tumours

Previous studies have shown the genes used in this study succumb to methylation in other malignancies such as certain forms of breast [24] and ovarian [37] cancer. Therefore, the reactivation described in this study may not be limited to colorectal cancer, but also other tumour types where reversal of epigenetic repression may be of therapeutic benefit. Efficacy of 5-aza-dC as a treatment is limited to certain tumour types, however, what causes a favourable outcome from 5-aza-dC treatment is unknown. Its success may lie with the methylation pattern at currently un-identified target genes and the drugs ability to reactivate silenced genes over a longer period of time. Therefore, our results suggest that repressed genes displaying localised TSS hypomethylation may be reactivated with combined methyltransferase/histone deacetylase inhibitor treatment. Increased acetylation at hypermethylated transcription start sites which lead to long term reactivation of anti-proliferative genes may be beneficial in the treatment of tumours when this information is known. This mechanism would be in addition to the reported synergistic apoptotic effect of methyltransferase and histone deacetylase inhibitors [28], [38].

### Conclusion

Analysis of the chromatin at the promoter of the genes in this study suggests that existing hypomethylation following (but not necessarily induced by) 5-aza-dC treatment, aids histone H3 acetylation, either directly or indirectly. The combination of hypomethylation of CpG sites at the TSS and H3 acetylation result in stable reactivation of the genes studied here. The results of this study highlight the epigenetic features which need to be modified with regard to the reversal of transcriptional status of genes in the treatment of disease. A more strategic approach will lead to the development of epigenetic therapies rather than the use of epigenetic-modifying drugs as cytotoxic therapies.

## Supporting Information

Figure S1 **Methylation of MAGEA3 in SW480 cells treated with 5-aza-dC.** Bisulfite sequencing PCR was performed at each time point and plotted to show changes before and after 5-aza-dC treatment, The MAGEA3 gene showed the greatest demethylation of all the genes assayed, with a decrease of 10–15% at several CpG sites near the TSS gene.
(TIF)

Figure S2 **Methylation at the MAGEA3 Transcription Start Site.** **A** - Promoter methylation across the MAGEA3 CpG island. The red bar indicates the region of sequence shown in B and C. **B** – Methylation at the MAGEA3 TSS in SW480 cells. Direct sequencing of bisulfite PCR products causes dual C and T peaks at CpG sites, and are representative of methylated and non-methylated alleles respectively. The CpG site adjacent to the TSS shows a greater proportion of T alleles (representing unmethylated cytosine) indicating hypomethylation, while nearby CpG sites show increased cytosine peaks and higher methylation levels. Arrows indicate CpG sites, boxed T's indicate position of non-CpG cytosine and underlined sequence represents the transcription start site. **C** – CpG sites in the HCT116 cell line are greater than 50% methylated.
(TIF)

Figure S3 **HCT116 chromatin changes at CDO1, HSPC105 and MAGEA3 transcription start sites.** Mock treatments are plotted with white data points and 5-aza-dC treated cells are plotted with black data points. The y-axis values represent percentage input, whilst x-axis values represent the four time-points of cell culture. Asterisks denote significant changes (t-test, p = <0.05) compared to the untreated point.
(TIF)

Figure S4 **SW480 chromatin changes at CDO1, HSPC105 and MAGEA3 transcription start sites.** Mock treatments are plotted with white data points and 5-aza-dC treated cells are plotted with black data points. The y-axis values represent percentage input, whilst x-axis values represent the four time-points of cell culture. Asterisks denote significant changes (t-test, p = <0.05) compared to the untreated point.
(TIF)

Figure S5 **HCT116 chromatin changes at CDKN2A, CXCL6 and ZFP3 transcription start sites.** Mock treatments are plotted with white data points and 5-aza-dC treated cells are plotted with black data points. The y-axis values represent percentage input, whilst x-axis values represent the four time-points of cell culture. Asterisks denote significant changes (t-test, p = <0.05) compared to the untreated point.
(TIF)

Figure S6 **SW480 chromatin changes at CDKN2A, CXCL6 and ZFP3 transcription start sites.** Mock treatments are plotted with white data points and 5-aza-dC treated cells are plotted with black data points. The y-axis values represent percentage input, whilst x-axis values represent the four time-points of cell culture. Asterisks denote significant changes (t-test, p = <0.05) compared to the untreated point.
(TIF)

Table S1 **Primer sequences used in Bisulfite PCR/sequencing and ChIP qPCR and expression qPCR.**
(DOC)
