# Supplementary figures and images for: Long Term Transcriptional Reactivation of Epigenetically Silenced Genes in Colorectal Cancer Cells Requires DNA Hypomethylation and Histone Acetylation
Source: PLoS One. 2011 Aug 4;6(8):e23127. doi: 10.1371/journal.pone.0023127 (PMC3150411; doi:10.1371/journal.pone.0023127)

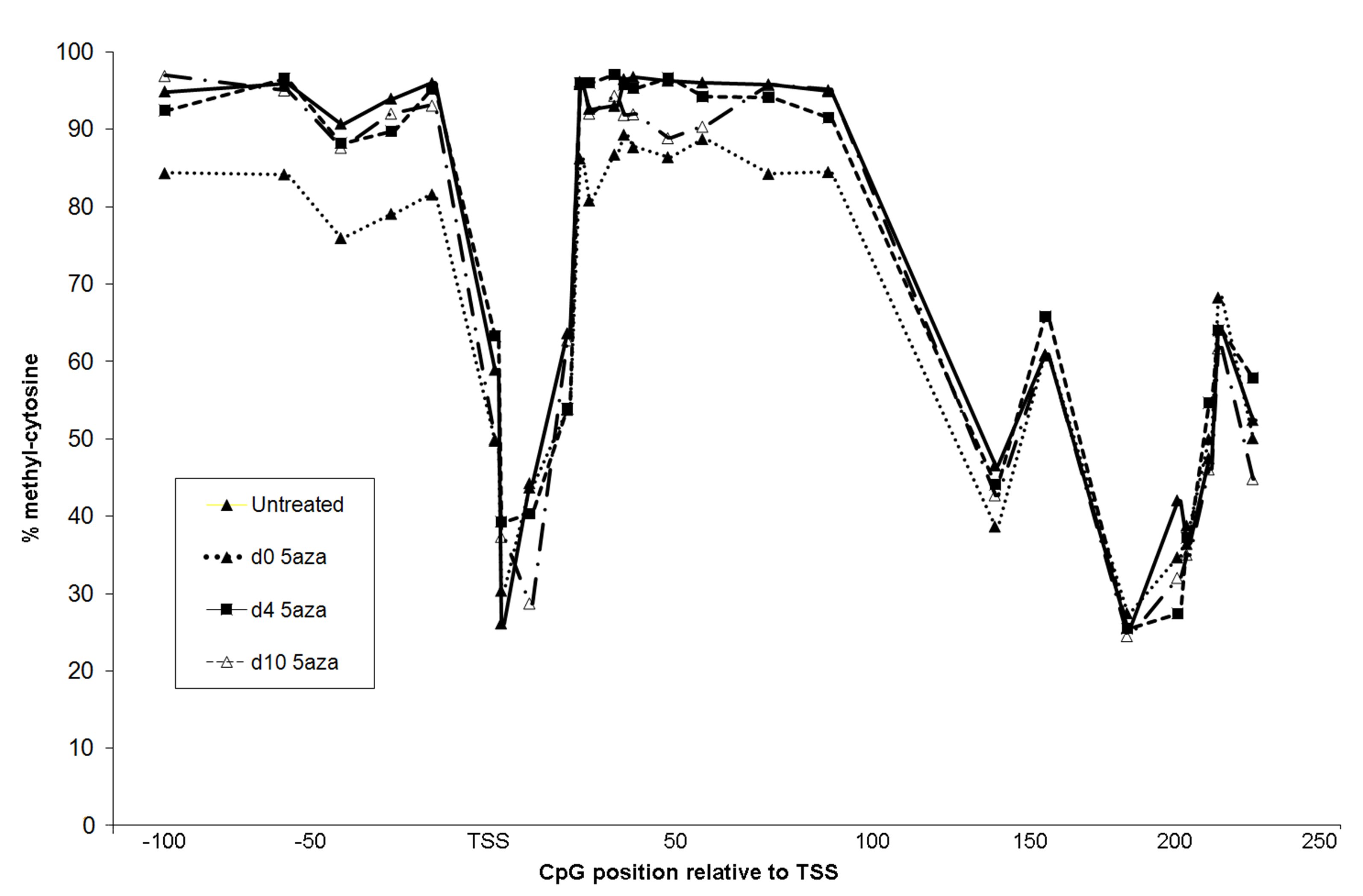

Supplement: Figure S1 — Methylation of MAGEA3 in SW480 cells treated with 5-aza-dC. Bisulfite sequencing PCR was performed at each time point and plotted to show changes before and after 5-aza-dC treatment, The MAGEA3 gene showed the greatest demethylation of all the genes assayed, with a decrease of 10–15% at several CpG sites near the TSS gene. (TIF) [file pone.0023127.s001.tif]

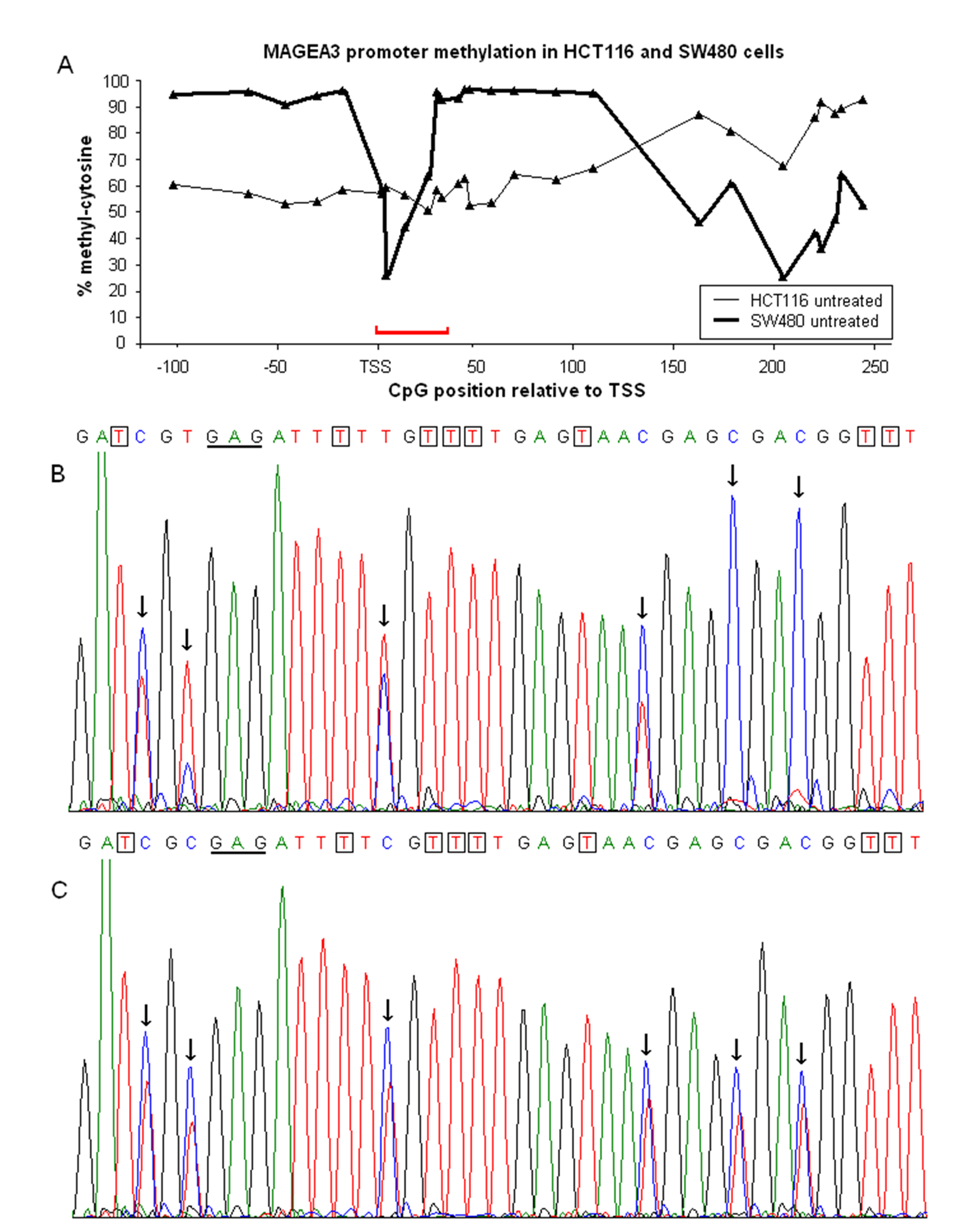

Supplement: Figure S2 — Methylation at the MAGEA3 Transcription Start Site. A - Promoter methylation across the MAGEA3 CpG island. The red bar indicates the region of sequence shown in B and C. B – Methylation at the MAGEA3 TSS in SW480 cells. Direct sequencing of bisulfite PCR products causes dual C and T peaks at CpG sites, and are representative of methylated and non-methylated alleles respectively. The CpG site adjacent to the TSS shows a greater proportion of T alleles (representing unmethylated cytosine) indicating hypomethylation, while nearby CpG sites show increased cytosine peaks and higher methylation levels. Arrows indicate CpG sites, boxed T's indicate position of non-CpG cytosine and underlined sequence represents the transcription start site. C – CpG sites in the HCT116 cell line are greater than 50% methylated. (TIF) [file pone.0023127.s002.tif]

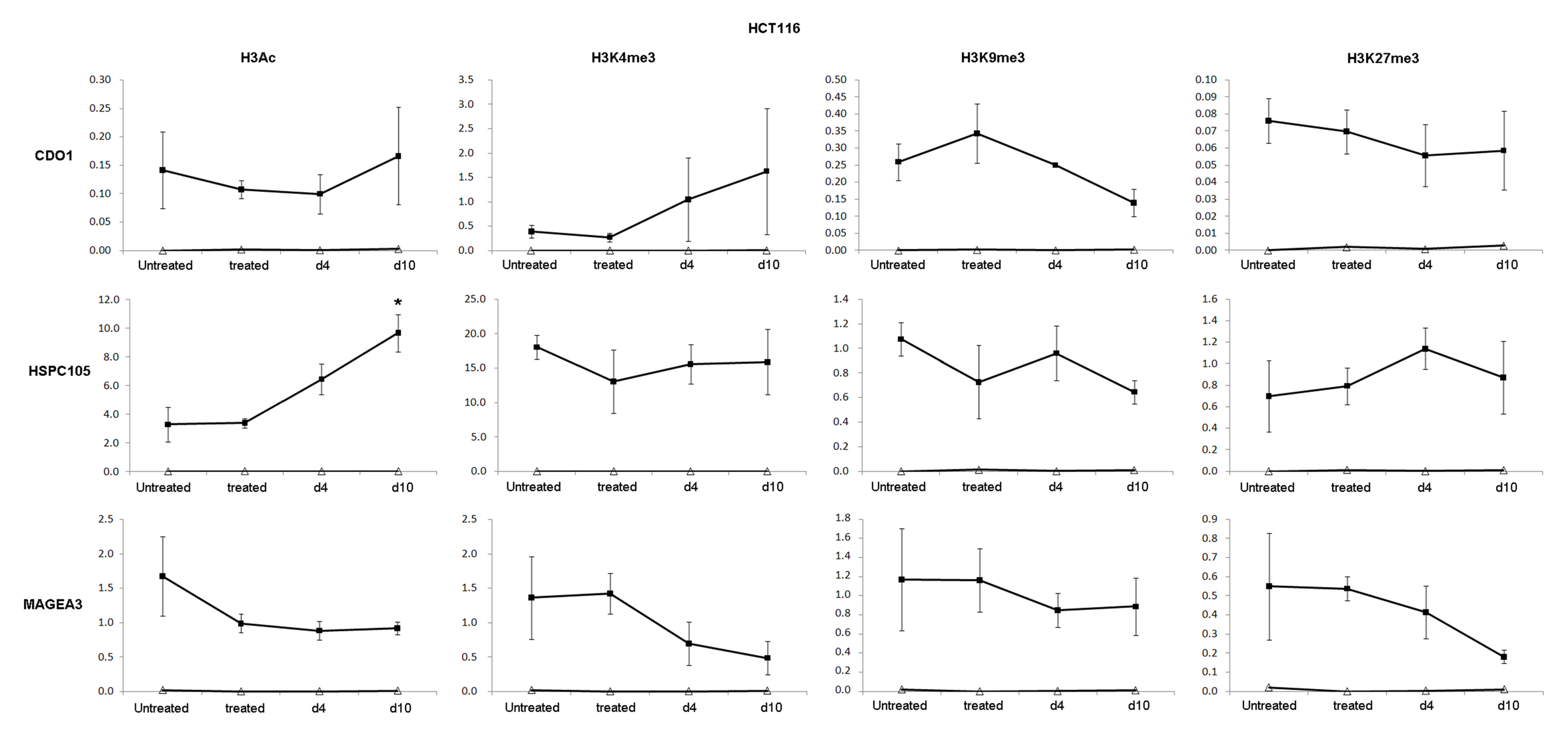

Supplement: Figure S3 — HCT116 chromatin changes at CDO1, HSPC105 and MAGEA3 transcription start sites. Mock treatments are plotted with white data points and 5-aza-dC treated cells are plotted with black data points. The y-axis values represent percentage input, whilst x-axis values represent the four time-points of cell culture. Asterisks denote significant changes (t-test, p = <0.05) compared to the untreated point. (TIF) [file pone.0023127.s003.tif]

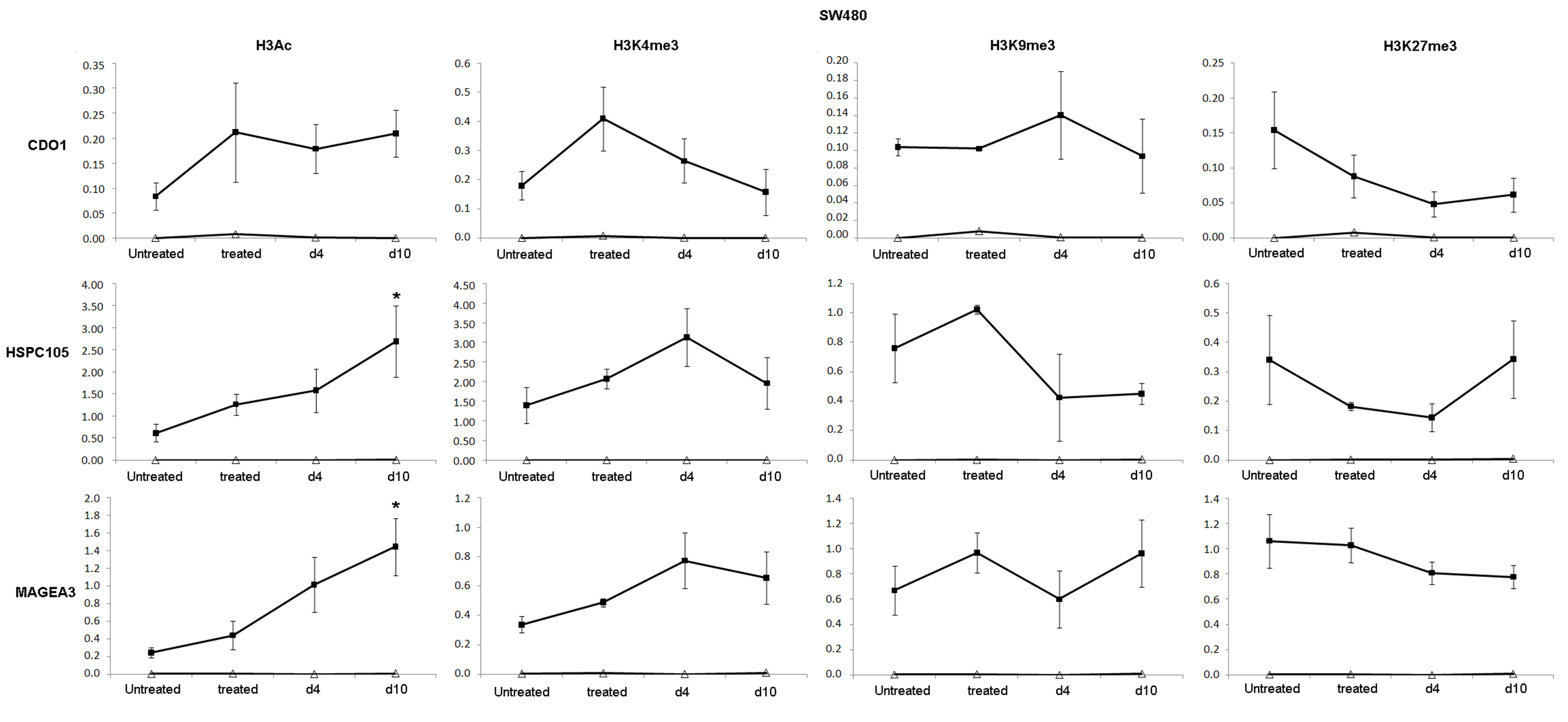

Supplement: Figure S4 — SW480 chromatin changes at CDO1, HSPC105 and MAGEA3 transcription start sites. Mock treatments are plotted with white data points and 5-aza-dC treated cells are plotted with black data points. The y-axis values represent percentage input, whilst x-axis values represent the four time-points of cell culture. Asterisks denote significant changes (t-test, p = <0.05) compared to the untreated point. (TIF) [file pone.0023127.s004.tif]

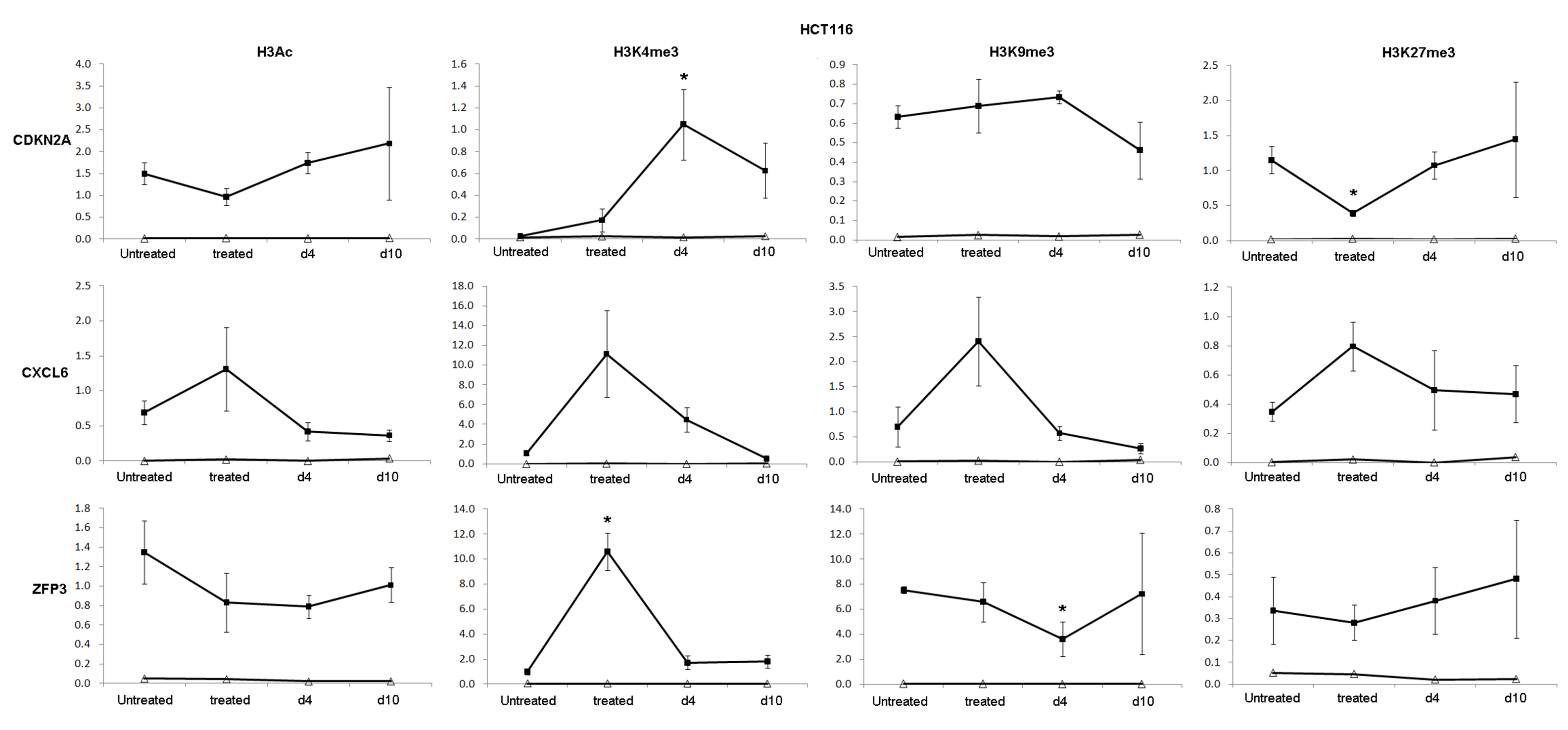

Supplement: Figure S5 — HCT116 chromatin changes at CDKN2A, CXCL6 and ZFP3 transcription start sites. Mock treatments are plotted with white data points and 5-aza-dC treated cells are plotted with black data points. The y-axis values represent percentage input, whilst x-axis values represent the four time-points of cell culture. Asterisks denote significant changes (t-test, p = <0.05) compared to the untreated point. (TIF) [file pone.0023127.s005.tif]

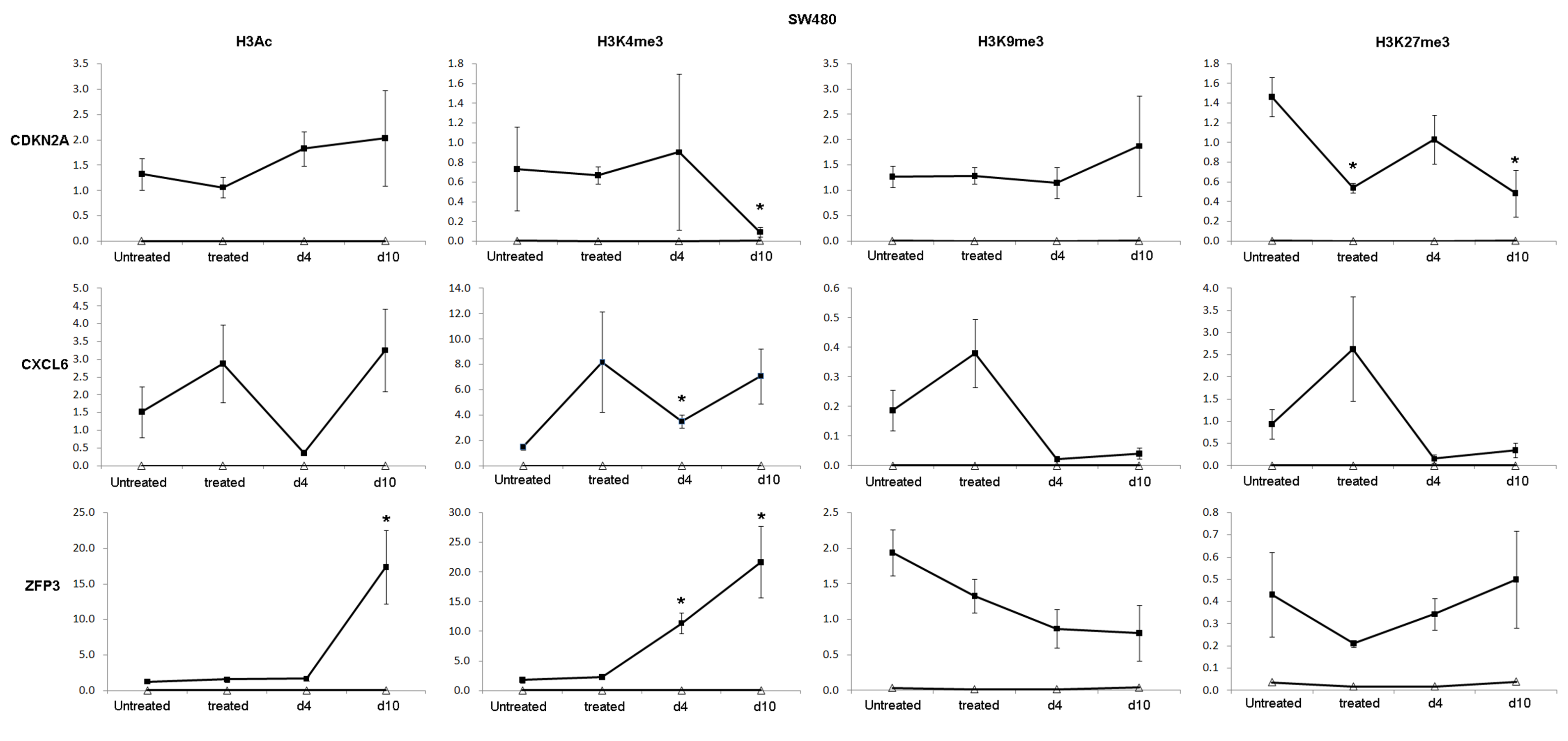

Supplement: Figure S6 — SW480 chromatin changes at CDKN2A, CXCL6 and ZFP3 transcription start sites. Mock treatments are plotted with white data points and 5-aza-dC treated cells are plotted with black data points. The y-axis values represent percentage input, whilst x-axis values represent the four time-points of cell culture. Asterisks denote significant changes (t-test, p = <0.05) compared to the untreated point. (TIF) [file pone.0023127.s006.tif]
