# Supplementary material for: Long Term Transcriptional Reactivation of Epigenetically Silenced Genes in Colorectal Cancer Cells Requires DNA Hypomethylation and Histone Acetylation
Source: PLoS One. 2011 Aug 4;6(8):e23127. doi: 10.1371/journal.pone.0023127 (PMC3150411; doi:10.1371/journal.pone.0023127)
Supplement: Table S1 — Primer sequences used in Bisulfite PCR/sequencing and ChIP qPCR and expression qPCR. (DOC) [file pone.0023127.s007.doc]

| **Gene (Accession Number)** | **Primer Direction** | **Bisulfite PCR and Sequencing (5’>3’)** | **ChIP (5’>3’)** | **Expression qPCR (5’>3’)** |
| --- | --- | --- | --- | --- |
|  |  |  |  |  |
| GAPDH (NM_002046.3) | Forward | GTTGGGATTGGTTGAGTT | TACTAGCGGTTTTACGGGCG | N/A |
|  | Reverse | CCAAACCTCCATACCCAAC | TCGAACAGGAGGAGCAGAGAGCGA | N/A |
|  |  |  |  |  |
| ACTB (NM_001101.2) | Forward | N/A | TGTGGCATCCACGAAACTACC | TGTGGCATCCACGAAACTACC |
|  | Reverse | N/A | ACATCTGCTGGAAGGTGGACA | ACATCTGCTGGAAGGTGGACA |
|  |  |  |  |  |
| CDO1 (NM_001801.2) | Forward | TTAAAGTGGGGGAGAGATTG | GAGGGAAAACCAGTGTGCCTAC | GGGAAAACCAGTGTGCCTACATC |
|  | Reverse | AACCTACACCTCCTCTACATTA | GCTCACAGCAGGTTCCGTATG | GTACAAGTGAAGGCTCACAGC |
|  |  |  |  |  |
| HSPC105 (NM_145168.2) | Forward | GTGAAAGTTTAAAAGTAGATAT | GTGTCCTCATTACAGGAGG | GTCTCCTCATTACAGGAGG |
|  | Reverse | CATTCTAAAAAACCAAACTAC | GCTTTCTCTACGTCAGACAGG | GCTTTCTCTACGTCAGACAGG |
|  |  |  |  |  |
| MAGEA3 (NM_005362.3) | Forward | GGATTTATAGTTTTAGGAT | ATCTGCCAGTGGGTCTCCATT | ATCTGCCAGTGGGTCTCCATT |
|  | Reverse | CACATTAAACTCTATCCCCAAAA | TCTGCTCAAGAGGCATGATGA | TCTGCTCAAGAGGCATGATGA |
|  |  |  |  |  |
| CXCL6 (NM_002993.3) | Forward | AGGGATGAATGTAGATAAAGGGAGTGT | AGCTCAGGAACCCGCGAAC | AACCCCAAAACGATTGGTAAACT |
|  | Reverse | CTTACACCACTTCCACCTTAA | CAGTGCCAGGAGCTCTCAC | GACAAACTTGCTTCCCGTTCTT |
|  |  |  |  |  |
| ZFP3 (NM_153018.1) | Forward | GAGTTTTTGAGTTTAGAGTAATGT | CTTCGGGCAGAGTTCTGAGC | CTTCGGGCAGAGTTCTGAGC |
|  | Reverse | CATAAACTTCAAAATCACACAAC | CTGAGTTCCCCCTGAAGGCC | CTGAGTTCCCCCTGAAGGCC |
|  |  |  |  |  |
| CDKN2A (NM_000077.3) | Forward | GATTTTAGGGGTGTTAT | GTCGGAGGCCGATCCAGGTCATG | GTCGGAGGCCGATCCAGGTCATGA |
|  | Reverse | CTCATTCCTCTTCCTTAAC | AGCGTGTCCAGGAAGCCCTC | AGCGTGTCCAGGAAGCCCTC |
|  |  |  |  |  |
| MLH1 (NM_000249.2) | Forward | AGATTATTTTAGTAGAGG | AGCTGATGGAAAGTGTGCATACA | AGCTGATGGAAAGTGTGCATACA |
|  | Reverse | AAAAAACCTAACTAACA | CGTGATCTGGGTCCCTTGA | CGTGATCTGGGTCCCTTGA |
|  |  |  |  |  |
| DICER1 (NM_030621.2) | Forward | N/A | N/A | CAAACCAGGTTGCTCAACAAG |
|  | Reverse | N/A | N/A | AACCTTGAGATCTGAATGAGTTCTGA |
